# Hepatocyte growth factor activator inhibitor-2 stabilizes Epcam and maintains epithelial organization in the mouse intestine

**DOI:** 10.1038/s42003-018-0255-8

**Published:** 2019-01-04

**Authors:** Makiko Kawaguchi, Koji Yamamoto, Naoki Takeda, Tsuyoshi Fukushima, Fumiki Yamashita, Katsuaki Sato, Kenichiro Kitamura, Yoshitaka Hippo, James W. Janetka, Hiroaki Kataoka

**Affiliations:** 1 Section of Oncopathology and Regenerative Biology, Department of Pathology, Faculty of Medicine, University of Miyazaki, Miyazaki 8891692, Japan; 2 Center for Animal Resources and Development, Kumamoto University, Kumamoto 8600811, Japan; 3 Division of Immunology, Department of Infectious Diseases, Faculty of Medicine, University of Miyazaki, Miyazaki 8891692, Japan; 4 Third Department of Internal Medicine, Interdisciplinary Graduate School of Medicine and Engineering, University of Yamanashi, 1110 Shimokato, Chuo, Yamanashi, 4093898 Japan; 5 Division of Molecular Carcinogenesis, Chiba Cancer Center Research Institute, Chiba 2608717, Japan; 6 Department of Medicine, Oncology Division, Washington University School of Medicine, 660 South Euclid Avenue, St. Louis, MO 63110 USA

## Abstract

Mutations in *SPINT2* encoding the epithelial serine protease inhibitor hepatocyte growth factor activator inhibitor-2 (HAI-2) are associated with congenital tufting enteropathy. However, the functions of HAI-2 in vivo are poorly understood. Here we used tamoxifen-induced Cre-LoxP recombination in mice to ablate *Spint2*. Mice lacking *Spint2* died within 6 days after initiating tamoxifen treatment and showed severe epithelial damage in the whole intestinal tracts, and, to a lesser extent, the extrahepatic bile duct. The intestinal epithelium showed enhanced exfoliation, villous atrophy, enterocyte tufts and elongated crypts. Organoid crypt culture indicated that *Spint2* ablation induced Epcam cleavage with decreased claudin-7 levels and resulted in organoid rupture. These organoid changes could be rescued by addition of serine protease inhibitors aprotinin, camostat mesilate and matriptase-selective α-ketobenzothiazole as well as by co-deletion of *Prss8*, encoding the serine protease prostasin. These results indicate that HAI-2 is an essential cellular inhibitor for maintaining intestinal epithelium architecture.

## Abstract (web summary)

Makiko Kawaguchi et al. developed an inducible *Spint2* knockout mouse model which exhibited extensive damage to the intestinal epithelium and resulted in death six days after tamoxifen-induced gene ablation. The extreme phenotype observed in this inducible line suggests an important role for *Spint2* in maintenance of healthy intestinal epithelium.

## Introduction

S*PINT2* encodes the type 1 transmembrane protein hepatocyte growth factor activator inhibitor (HAI)-2 that has two extracellular Kunitz-type serine protease inhibitor domains, namely KD1 (N-terminal side Kunitz domain) and KD2 (C-terminal side Kunitz domain), a single path transmembrane domain and a short intracytoplasmic domain^1,2^. The protease inhibitor domain of HAI-2 is homologous to that of HAI-1. HAI-2 and HAI-1 also have a similar anti-protease spectrum and are frequently co-expressed by epithelial cells. They regulate the activities of serum hepatocyte growth factor activator (HGFA), cellular type 2 transmembrane serine proteases (TTSP), particularly matriptase, and glycosylphosphatidylinositol-anchored serine protease, Prss8 (also known as prostasin)^1,3–5^. However, in a human cell line expressing both HAI-1 and HAI-2, HAI-2 could not compensate for loss of HAI-1 and vice versa, and silencing of *SPINT1* or *SPINT2* produces specific and differing phenotypes, suggesting that these two protease inhibitors have distinct roles in epithelial cells^1,6,7^. Indeed, HAI-1 has a distinct cell surface localization, whereas HAI-2 localizes to the cytoplasm^8–10^. In mice, deletion of either *Spint1* or *Spint2* produces an embryonic lethal phenotype, also indicating that Hai-2 does not compensate for loss of Hai-1 and vice versa in vivo^11,12^.

Mutations in the *SPINT2* gene cause a syndromic form of congenital sodium diarrhea (CSD), a rare autosomal-recessive disorder that occurs during infancy^13^, suggesting a fundamental role for HAI-2 in the function of intestinal mucosa. Syndromic CSD is associated with choanal or anal atresia, hypertelorism, and corneal erosions. Five distinct mutations of the *SPINT2* gene have been reported for syndromic CSD, and the mutations associated with this disease result in either reduced protease inhibitor activity of HAI-2 or loss of HAI-2 synthesis^13–15^. Notably, a single amino acid mutation in KD2 (Y163C) is sufficient to induce syndromic CSD, suggesting that KD2 is critical for HAI-2 function in the intestine. For histopathological classification, *SPINT2* mutation-related enteropathy is classified as congenital tufted enteropathy (CTE), characterized by villous atrophy and focal crowding at the villus tips due to disorganization of enterocytes^15,16^. Given that conventional CTE is caused by mutations in the *EPCAM* gene that encodes epithelial cell adhesion molecule (EpCAM)^15^, *SPINT2* mutation-induced enteropathy may arise from enhanced EpCAM cleavage induced by dysregulated matriptase due to insufficient HAI-2 function, as suggested in a recent in vitro study by Wu et al.^17^.

*Spint2* knockout mice are embryonic lethal, and show embryonic ectoderm clefting, defects in neural tube closure, and impaired development of the placental labyrinth^12^. However, there are few reports that analyzed the in vivo molecular functions of HAI-2/Hai-2. Szabo et al.^18^ showed that crossbreeding *Spint2* null mice with Prss8 (also known as prostasin) hypomorphic mutant mice rescued the embryonic lethal phenotype associated with *Spint2* deficiency^19^. Analysis of intestinal epithelium of double mutant neonates indicated that Hai-2 loss results in abnormal localization and activation of matriptase through Prss8 dysregulation^4^. Those neonates shows histological changes of the intestine reminiscent to CTE and die within 4 to 7 days after birth^19^. However, the *Spint2*-*Prss8* double mutant mouse model is insufficient for use in studies to clarify the role of Hai-2 in normal homeostasis and disease in vivo when wild-type Prss8 is present. Therefore, a conditional knockout mouse system for *Spint2* is required to analyze the in vivo physiological and pathophysiological roles of HAI-2/Hai-2 in detail.

In this report we describe the generation of a conditional *Spint2* knockout mouse model based on the Cre recombinase and LoxP system. Using a tamoxifen-inducible Cre recombinase (CreERT2) expression system, we examined the effect of spontaneous *Spint2* deletion on mice 6 weeks after birth. We found that Hai-2 is an essential protease inhibitor for epithelial integrity, Epcam homeostasis and mucosal organization of the intestinal tract.

## Results

### Lethal effect of spontaneous Hai-2 loss in mice

**Fig. 1:**
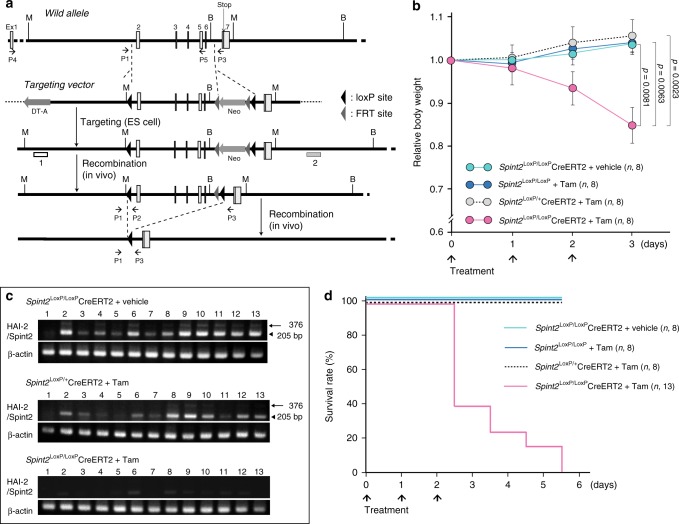
Generation of conditional *Spint2* knockout mice and effect of spontaneous Hai-2 loss on survival. **a** Schematic representation of the *Spint2* gene, targeting vector, floxed *Spint2* gene, and floxed *Spint2* gene after Cre-mediated recombination. The recombination results in excision of exons 2 to 6. M, *Mfe*I site; B, *Bam*HI site. 1 and 2: Southern probe. Positions of PCR primers P1, P2, and P3 for genotyping and P4 and P5 for RT-PCR are also shown. **b** The effect of *Spint2* deletion on body weight gain in mice. Six-week-old male mice were treated with tamoxifen in corn oil (Tam) or corn oil alone (vehicle) for 3 days. Arrows indicate treatment points. The number of mice in each group is also indicated (*n*). Data are mean ± standard error (SE). *p-*value; two-way repeated-measures ANOVA. **c** Representative RT-PCR for Hai-2 mRNA in *Spint2*^LoxP/LoxP^Cre-ERT2 mice given a daily intraperitoneal injection of Tam or vehicle for 3 days. Arrowhead and arrow indicate major Hai-2 isoform of mice carrying only KD2 and the minor isoform including both KD1 and KD2, respectively^27^. 1, brain; 2, thymus; 3, lungs; 4, liver; 5, spleen; 6, kidneys; 7, forestomach; 8, glandular stomach; 9, jejunum; 10, ileum; 11, small intestinal mucosa; 12, cecum; 13, colon. **d** Effect of *Spint2* deletion on survival. Six-week-old male mice were treated with Tam or vehicle for 3 days. Arrows indicate treatment points. The mice were observed every 12 h and survival was plotted for each *Spint2*^LoxP/LoxP^ mouse treated with Tam (i.e., wild-type *Spint2*), *Spint2*^LoxP/LoxP^CreERT2 treated with vehicle only (wild-type *Spint2*), *Spint2*^LoxP/+^CreERT2 treated with Tam (*Spint2* heterozygous deletion), or *Spint2*^LoxP/LoxP^CreERT2 treated with Tam (*Spint2* homozygous deletion). The number of mice in each group is also indicated (*n*)

To circumvent the embryonic lethality associated with Hai-2 deficiency, we engineered mice homozygous for *Spint2* floxed alleles in which Cre recombinase expression results in the deletion of most of the *Spint2* gene coding region, including exons encoding KD1 (exon 2) and KD2 (exon 5) (Fig. 1a and Supplementary Fig. 1a). The floxed mice were then crossed with ROSA26-CreERT2 mice to generate *Spint2*^LoxP/LoxP^CreERT2 mice. We then analyzed the effects of *Spint2* deletion following intraperitoneal administration of tamoxifen to *Spint2*^LoxP/LoxP^CreERT2 mice (homozygous *Spint2* deletion) at 6 weeks of age to activate the Cre recombinase and compared the induced phenotypes with those of vehicle-treated controls (wild-type *Spint2*) and also with tamoxifen-treated *Spint2*^LoxP/+^CreERT2 mice (heterozygous *Spint2* deletion). Within three days, tamoxifen-treated *Spint2*^LoxP/LoxP^CreERT2 mice were lethargic and had significant weight loss (Fig. 1b). Three days after starting tamoxifen treatment, Hai-2 mRNA was hardly detectable in major Hai-2-expressing organs such as the intestinal tract (Fig. 1c). Genomic polymerase chain reaction (PCR) confirmed the DNA rearrangement in all tissues except for the brain (Supplementary Fig. 1b). Notably, the *Spint2*^LoxP/LoxP^CreERT2 mice survived no more than 6 days after beginning tamoxifen treatment, indicating that the induced loss of Hai-2 protein had a lethal effect (Fig. 1d). Similar results were seen for another *Spint2*^LoxP/LoxP^ mouse line derived from an independently targeted embryonic stem (ES) clone (ES89; Supplementary Table 1).

### Hai-2 loss induced severe epithelial damage of the intestine

**Fig. 2:**
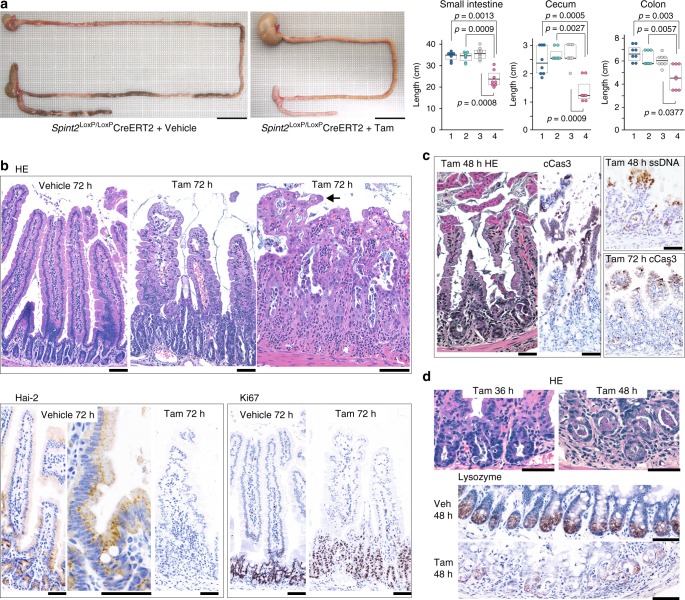
Effects of *Spint2* deletion on the gastrointestinal tract. **a** Decreased intestinal length in *Spint2*-deleted mice. Representative macroscopic photos of gastrointestinal tissue (left; bars, 2 cm) and box plots of intestinal length (right) are shown. 1, *Spint2*^LoxP/LoxP^ mice treated with tamoxifen (Tam); 2, *Spint2*^LoxP/LoxP^CreERT2 mice treated with corn oil only (vehicle); 3, *Spint2*^LoxP/+^CreERT2 mice treated with Tam; 4, *Spint2*^LoxP/LoxP^CreERT2 mice treated with Tam. The 25th and 75th percentile (boxes) and the median (bold line within the boxes) are plotted. Circle represents the value of each case. *N* = 8 for each group. *p-*value; Mann–Whitney *U-*test. **b** Histology of small intestine (*Spint2*^LoxP/LoxP^CreERT2 mice) tissue 72 h after tamoxifen or vehicle treatment. Representative photos of HE-stained sections from the proximal small intestine of the vehicle-treated mouse (upper panel, left), proximal small intestine of the tamoxifen-treated mouse (center) and distal small intestine of the tamoxifen-treated mouse (right) are shown. Arrow indicates a tuft formed by enterocytes. Photos of Hai-2 and Ki67 immunohistochemistry are also shown in the lower panel. Bars, 50 μm. **c** Detachment of surface epithelial sheets observed 48 h after tamoxifen treatment. Representative photos of HE-stained and cleaved caspase-3 (cCas3)-stained section from the proximal small intestine (left panel) are shown. Distal small intestine showed similar change and the cells were also immunohistochemically positive for ssDNA (right upper panel). At 72 h after tamoxifen treatment, villi were shortened with many cCas3-positive cells in the upper portion and increased numbers of apoptotic bodies in the crypts (proximal small intestine; right lower panel). Bars, 50 μm. **d** Decreased numbers of Paneth cells in the small intestine. HE staining and immunohistochemical staining for lysozyme are shown. Bars, 50 μm

To clarify the cause of lethality in *Spint2*^LoxP/LoxP^CreERT2 mice, we sacrificed the mice 24, 36, 48, and 72 h after starting tamoxifen treatment. At 72 h, macroscopic phenotypes were observed in the gastrointestinal tracts of tamoxifen-treated *Spint2*^LoxP/LoxP^CreERT2 mice, but not in other organs. In the treated mice, the length of the intestine was shorter compared to the control mice and the tissue showed diffuse mucosal damage. The stomach was expanded (Fig. 2a). Subsequent histopathological evaluation revealed successful ablation of the Hai-2 protein and diffuse epithelial damage with mucosal disorganization throughout the intestinal tracts of *Spint2*^LoxP/LoxP^CreERT2 mice. In the small intestine, 72 h after starting the tamoxifen treatment *Spint2* inactivation dramatically altered the mucosal architecture as evidenced by severe villus atrophy and significantly elongated crypts that showed proliferative activity (Fig. 2b). The ratio of villous length relative to the corresponding crypt depth was significantly decreased in tamoxifen-treated mice relative to vehicle-treated control mice (*p* *=* 0.0005) (Supplementary Fig. 2a). In the villous epithelium, stacking of enterocyte nuclei produced a tufted appearance compatible with descriptions of intestinal specimens from CTE patients (Fig. 2b)^15,16^. The time-course observation also indicated that the number of enterocytes positive for cleaved caspase-3 increased in the upper portion of the crypts and villus base 36 h after tamoxifen treatment (Supplementary Fig. 2b). After 48 h, increased exfoliation of single epithelial cells was present, with peeling of enterocyte sheets from the villous tip (Fig. 2c). These peeling cells were positive for staining with antibodies against cleaved caspase-3 and single-strand DNA (ssDNA), suggestive of apoptotic changes (Fig. 2c). Meanwhile, the number of Paneth cells decreased in the crypt base (Fig. 2d) in a statistically significant level (*p* = 0.0028, Student *t*-test, Supplementary Fig. 2c).

**Fig. 3:**
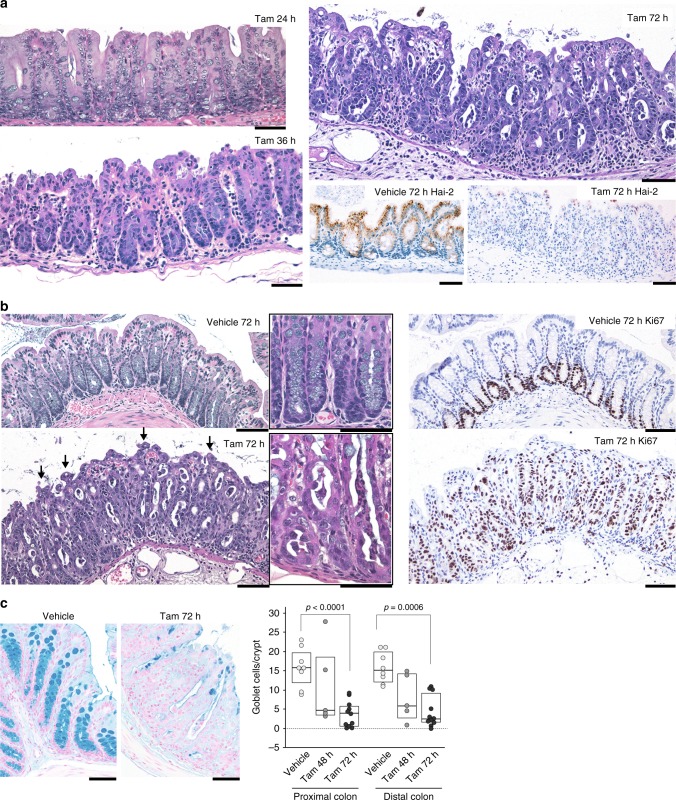
Effects of Hai-2 ablation on large intestine. **a** Histology of the cecum from *Spint2*^LoxP/LoxP^CreERT2 mice after tamoxifen or vehicle (corn oil) treatment. Representative photos of HE from mice 24, 36, and 72 h after tamoxifen treatment are shown. Successful Hai-2 ablation was confirmed by immunohistochemistry. **b** Histology of the colon 72 h after tamoxifen treatment. Crypts were elongated with many exfoliating, degenerated cells present in the lumen. Arrows indicate a tuft formed by enterocytes. Higher magnification images are shown in the insets. Representative photos of Ki67 immunohistochemistry are also presented (right panel). **c** Decreased numbers of goblet cell in *Spint2*-deleted colon mucosa. Alcian-blue staining. The 25th and 75th percentile (boxes) and the median (bold line within the boxes) are plotted. Circle represents the value of each case. Goblet cells per crypt of *Spint2*-deleted mice (*n* = 5 and 11 for 48 h and 72 h after starting treatment, respectively) in the proximal and distal colon were counted and compared to that for vehicle-treated control mice (*n* = 8). *p-*value; Mann–Whitney *U-*test. Bars, 50 μm (**a**–**c**)

Diffuse epithelial damage was also evident in the large intestine. Both the cecum and colon showed obvious, diffuse epithelial damage 36 h after treatment, with increased numbers of apoptotic bodies and exfoliating cells (Fig. 3a). The number of crypts was decreased by 48 h, followed by an active regeneration phase when elongated crypts became apparent. After 72 h, mucosal disorganization was evident, showing marked mucosal thickening with elongated crypts, reactive epithelial cell atypia, and small tufted structures consisting of stacked enterocytes on the surface (Fig. 3a, b). The elongated crypts were composed mostly of Ki67-positive proliferating cells, and many detached cells having pyknotic nuclei were also present (Fig. 3b). The number of goblet cells showed a time-dependent decrease after tamoxifen treatment (Fig. 3c). Collectively, the absence of Hai-2 proteins impaired the terminal differentiation capacity needed to establish a normal epithelial structure. However, no increase in the frequency of β-catenin nuclear translocation was observed during the observation period, indicating that abnormal Wnt/β-catenin signaling did not contribute to the Hai-2 loss-induced enteropathy (Supplementary Fig. 2d).

Although the glandular epithelium of the stomach also showed slightly increased amounts of apoptosis and focal erosion, these changes were not as pronounced as those seen in the intestine (Supplementary Fig. 3). Thus, the marked stomach distention was likely secondary to intestinal dysfunction. Taken together, the marked structural damages and dysfunction of the intestinal epithelium likely underpin the lethality caused by spontaneous loss of Hai-2 in mice.

### Extrahepatic biliary tracts were also affected by Hai-2 loss

**Fig. 4:**
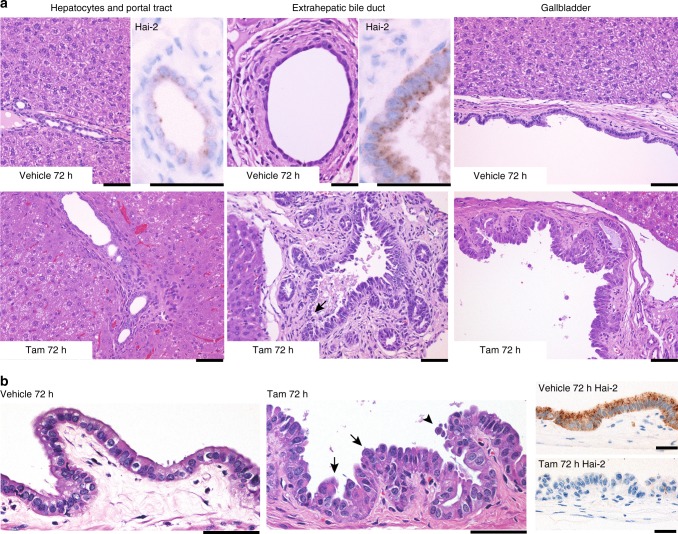
Histology of biliary tracts of *Spint2*^LoxP/LoxP^CreERT2 mice 72 h after tamoxifen or vehicle (corn oil) treatment. **a** Histology of interlobular small bile duct (left panel), extrahepatic bile duct (center) or gallbladder (right). Hai-2 ablation resulted in severe morphological alterations of the extrahepatic biliary epithelium. Arrow indicates erosion observed in Hai-2-deficient bile duct mucosa. Higher magnification photos of Hai-2 immunohistochemistry of bile ducts from vehicle-treated mice are also shown. Bars, 50 μm. **b** Higher magnification of gallbladder epithelium. Arrows and arrowhead indicate epithelial tufts and exfoliating cells, respectively. Representative images of Hai-2 immunohistochemistry are also shown. Bars, 50 μm

As HAI-2/Hai-2 is widely expressed in epithelial tissues in humans and mice^1,2,20^, we also searched for additional histopathological changes in other epithelial tissues. Significant alterations were observed in the epithelium of the gallbladder and extrahepatic bile duct of *Spint2*^LoxP/LoxP^CreERT2 mice. The gallbladder epithelial cells tended to have a hobnail appearance, occasionally forming a slight tufted structure and increased exfoliation (Fig. 4a). However, apoptotic bodies were not evident compared with that seen in the intestinal epithelium. Although the epithelium of the extrahepatic biliary tract showed similar changes, the intrahepatic small bile ducts and pancreatic duct were not notably affected by Hai-2 ablation (Fig. 4b and Supplementary Fig. 3). In accordance with this observation, Hai-2 expression levels were lower in the intrahepatic small bile ducts and pancreatic ducts compared with the epithelia of gallbladder and extrahepatic bile duct (Fig. 4). The skin, bronchopulmonary epithelial cells, esophageal epithelium, and renal tubules showed no obvious histopathological changes during the observation period (Supplementary Fig. 3).

### Hai-2 loss led to destruction of intestinal organoids

**Fig. 5:**
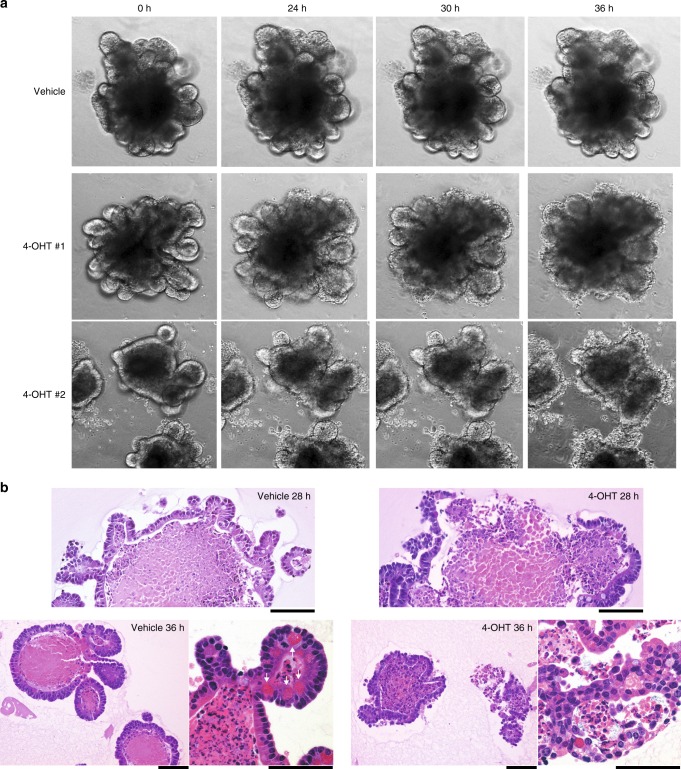
Organoid crypt culture of small intestine from *Spint2*^LoxP/LoxP^CreERT2 mice treated with (4-OHT) or without (vehicle) 1 μM 4-OHT. **a** Representative photos of phase-contrast microscopy of time-course for changes of organoids treated with 1 μM 4-OHT (two independent experiments) or vehicle (ethanol) only. **b** HE staining of organoids 28 h (upper panel) and 36 h (lower panel) after treatment. Arrows indicate Paneth cells. Bars, 50 μm

Epithelial damage and disorganization of the intestinal architecture in *Spint2*-deleted mice could be a direct effect of Hai-2 loss in epithelial cells or arise from secondary stromal inflammation caused by leaky epithelia and intestinal microbial colonization. To distinguish between these possibilities, we performed in vitro three-dimensional (3-D) crypt organoid culture of *Spint2*^LoxP/LoxP^CreERT2 small intestine cells. Similar to in vivo observations, deterioration of organoid integrity began 24–28 h after treatment with 4-hydroxytamoxifen (4-OHT) (Fig. 5a and Supplementary Movies 1 and 2). At 36 h (Fig. 5b), destruction or rupture of crypt structure was apparent (Supplementary Movies 1 and 2), whereas the vehicle-treated organoids were intact (Supplementary Movie 3).

### Disruption of Epcam/claudin-7 complex by Hai-2 loss

**Fig. 6:**
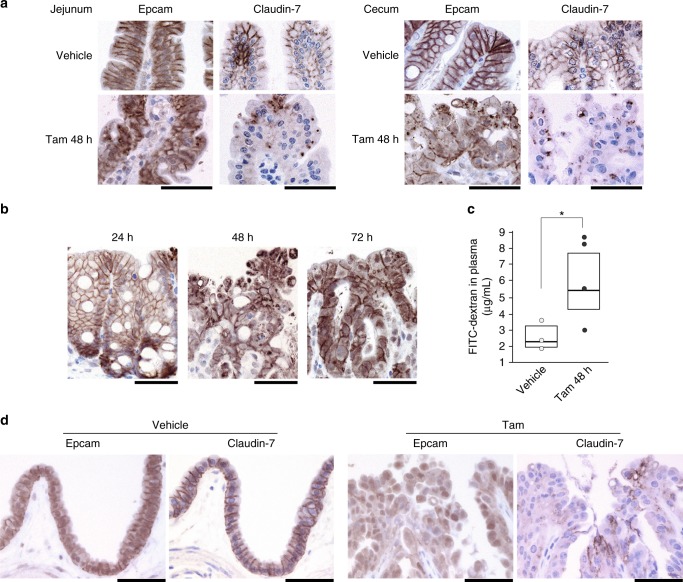
Disruption of Epcam and claudin-7 after Hai-2 ablation and increased mucosal permeability in vivo. **a** Immunohistochemical analysis of Epcam and claudin-7 in the jejunum (villus portion) and cecum 48 h after tamoxifen (Tam) treatment in *Spint2*^LoxP/LoxP^CreERT2 mice. Epithelial cell surface localization of Epcam and claudin-7 was severely disrupted by *Spint2* deletion. **b** Immunohistochemical analysis of Epcam in the cecum 24, 48, and 72 h after tamoxifen treatment. **c** Mucosal permeability in *Spint2*^LoxP/LoxP^CreERT2 mice 48 h after vehicle (corn oil) (*n*, 3) and tamoxifen (*n*, 4) treatment. The 25th and 75th percentile (boxes) and the median (bold line within the boxes) are plotted. Circle represents the value of each case. *p = 0.00034 (Student *t*-test). **d** Immunohistochemical analysis of Epcam and claudin-7 in the gallbladder epithelium 72 h after vehicle or tamoxifen (Tam) treatment in *Spint2*^LoxP/LoxP^CreERT2 mice. Bars, 50 μm (**a**, **b**, and **d**)

CSD patients, including those with syndromic-form CSD caused by *SPINT2* mutation, show intestinal histology compatible with that observed in CTE caused by *EPCAM* mutations. Therefore, we performed immunohistochemical analysis of the expression and localization of Epcam and one of its essential partners in the intestinal epithelium, claudin-7^21,22^. Hai-2-deficient epithelium showed decreased cell surface Epcam immunoreactivity and mislocalization within the cytoplasm of disorganized epithelial cells, particularly in cells forming tufts, as well as accompanying decreases in claudin-7 immunoreactivity (Fig. 6a). The disruption and mislocalization of Epcam peaked 48 h after tamoxifen treatment, as did increases in mucosal permeability (Fig. 6b, c). Loss of cell surface Epcam and claudin-7 immunoreactivity was also observed in Hai-2-deficient extrahepatic biliary epithelium (Fig. 6d).

**Fig. 7:**
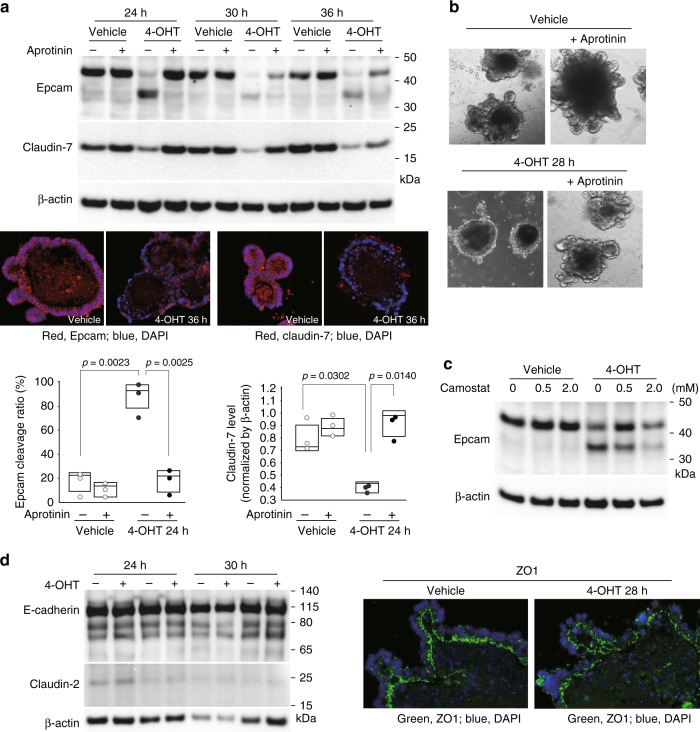
Enhanced cleavage of Epcam in response to Hai-2 ablation. **a** Immunoblot analysis of Epcam and claudin-7 in intestinal organoids from *Spint2*^LoxP/LoxP^CreERT2 mice. Organoids were treated with 4-OHT (1 μM) or vehicle only (ethanol) for the indicated time with or without aprotinin (200 μg/mL). 4-OHT-induced ablation of Hai-2 resulted in Epcam cleavage accompanied by significantly decreased intensity of claudin-7 bands; this effect was rescued completely by aprotinin. The 25th and 75th percentile (boxes) and the median (bold line within the boxes) are plotted. Circle represents the value of each case. *p-*value; Student *t*-test. **b** Effect of aprotinin (200 μg/mL) on Hai-2 ablation-induced organoid destruction (28 h after 4-OHT treatment). **c** Dose-dependent effects of camostat mesilate on Epcam cleavage 24 h after 4-OHT treatment. **d** Effects of Hai-2 ablation on E-cadherin, claudin-2 (immunoblot, left panel) and ZO-1 (immunofluorescence, right panel)

We then analyzed Epcam and claudin-7 expression in 3-D intestinal organoid cultures from *Spint2*^LoxP/LoxP^CreERT2 mice. Immunoblot analysis showed enhanced levels of a 35-kDa cleavage product of Epcam in the absence of Hai-2 as well as markedly decreased claudin-7 levels (Fig. 7a). Immunofluorescence analysis of these proteins also showed significantly decreased cell surface Epcam and claudin-7 in organoids after 4-OHT treatment (Fig. 7a). On the other hand, Epcam and claudin-7 mRNA levels were not affected (Supplementary Fig. 4), indicating that the decrease in cell surface Epcam and claudin-7 in response to *Spint2*-deletion occurred at a post-translational level. Importantly, addition of the protease inhibitor aprotinin to the culture media significantly suppressed Epcam cleavage and concomitant decreases in claudin-7 levels (Fig. 7a). Therefore, an excess of trypsin-like serine protease activity was likely responsible for Epcam cleavage. Notably, aprotinin rescued Hai-2 ablation-induced destruction in organoids (Fig. 7b). Similar in vitro results were also obtained following the addition of the synthetic serine protease inhibitor camostat mesilate (Fig. 7c). However, in vivo administration of camostat mesilate (oral or intraperitoneal) could not rescue the lethal phenotype of *Spint2*^LoxP/LoxP^CreERT2 mice after tamoxifen treatment (Supplementary Fig. 5). The ineffectiveness of camostat mesilate in vivo may be due to insufficient concentration of the inhibitor in the intestinal microenvironment in vivo, which will require further studies.

There were no significant changes in the expression of other junctional proteins, including E-cadherin, claudin-2 and ZO-1 in response to Hai-2 ablation (Fig. 7d). Taken together, these results indicate that Hai-2 is an essential cellular protease inhibitor needed to stabilize the Epcam/claudin-7 complex in the intestine.

### Prss8 was required for Epcam cleavage in *Spint2*^−/−^ organoids

**Fig. 8:**
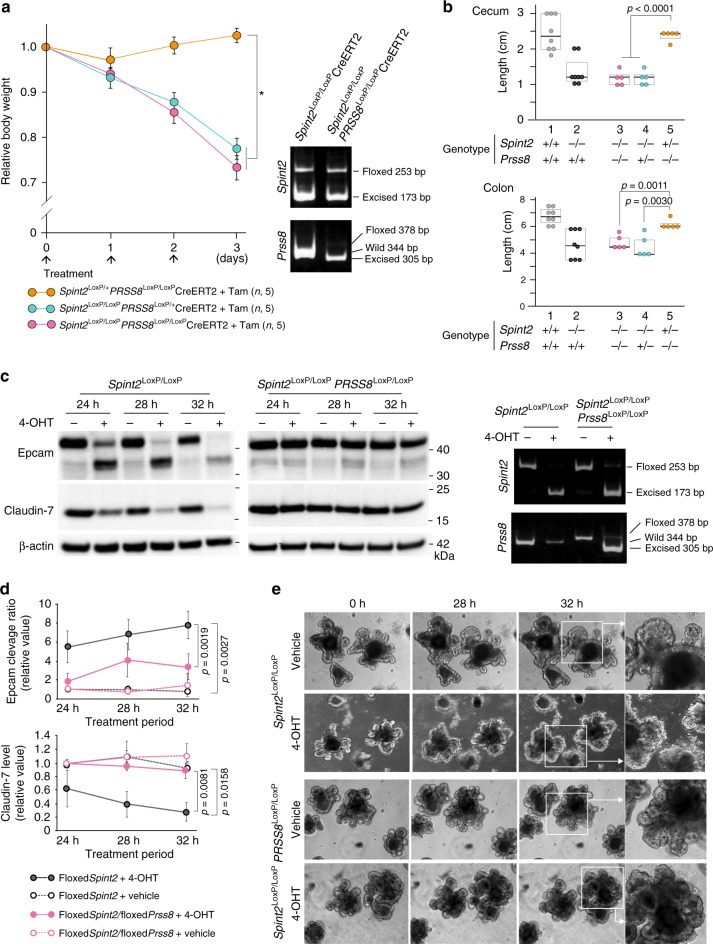
Effect of *Prss8* co-deletion on spontaneous *Spint2* deletion-induced phenotype in mice. **a** The effect of tamoxifen-induced *Prss8* co-deletion on body weight gain in mice with homozygous or heterozygous *Spint2* deletion. Six-week-old male mice were treated with tamoxifen (Tam) or vehicle for 3 days. Arrows indicate treatment points. The number of mice in each group is also indicated (*n*). Data are mean ± SE. *p < 0.0001 (two-way repeated-measures ANOVA). Representative results of genotyping of small intestine 72 h after starting Tam treatment are also shown. **b** Effect of *Prss8* co-deletion on cecum and colon length 72 h after tamoxifen treatment. 1, *Spint2*^LoxP/LoxP^ (*n*, 8); 2, *Spint2*^LoxP/LoxP^CreERT2 (*n*, 8); 3, *Spint2*^LoxP/LoxP^*Prss8*^LoxP/LoxP^CreERT2 (*n*, 5); 4, *Spint2*^LoxP/LoxP^*Prss8*^LoxP/+^CreERT2 (*n*, 5); 5, *Spint2*^LoxP/+^*Prss8*^LoxP/LoxP^CreERT2 (*n*, 5). Box plots are shown and the median is indicated by a bold vertical line. Circle represents the value of each case. *p-*value: Student *t*-test. The data for 1 and 2 are from Fig. 2a. Genotypes of each group after tamoxifen treatment are also indicated. **c** Time-course of Epcam cleavage and claudin-7 band intensity of organoids from *Spint2*^LoxP/LoxP^*Prss8*^LoxP/LoxP^CreERT2 mice and littermate *Spint2*^LoxP/LoxP^CreERT2 mice. Hai-2 ablation-induced Epcam cleavage and claudin-7 loss were alleviated by Prss8 ablation. Data of genotyping at 28 h after starting 4-OHT treatment (1 μM) are indicated in the right panel. **d** Time-course quantitative analysis of Epcam cleavage and claudin-7 levels after 4-OHT (1 μM) treatment of the organoids from *Spint2*^LoxP/LoxP^CreERT2 (floxed*Spint2*) and *Spint2*^LoxP/LoxP^*Prss8*^LoxP/LoxP^CreERT2 (floxed*Spint2*/floxed*Prss8*) mice. Data are mean ± SE. *p-*value: Student *t*-test, *n* = 3. **e** Time-course morphology of organoids from organoid of small intestine from *Spint2*^LoxP/LoxP^CreERT2 or *Spint2*^LoxP/LoxP^*Prss8*^LoxP/LoxP^CreERT2 mice treated with or without (vehicle) 1 μM 4-OHT

In mouse studies, embryonic lethality of *Spint2*^−/−^ mice could be prevented by reducing Prss8 (prostasin) activity^4,18,19^. In the human intestinal epithelial cell line Caco2, HAI-2 regulates PRSS8, which is required for normal cell surface localization and function of matriptase^4,23^. Moreover, in these cells, HAI-2 insufficiency leads to enhanced EpCAM cleavage by matriptase^17^. These lines of evidence suggest that phenotypes induced by spontaneous ablation of Hai-2 may be secondary to dysregulation of the Prss8-matriptase axis. To explore this possibility, we crossed *Spint2*^LoxP/+^CreERT2 mice with *Prss8*^LoxP/+^ mice to generate either *Spint2*^LoxP/LoxP^*Prss8*^LoxP/+^CreERT2, *Spint2*^LoxP/+^*Prss8*^LoxP/LoxP^CreERT2 or *Spint2*^LoxP/LoxP^*Prss8*^LoxP/LoxP^CreERT2 mice, which were treated with tamoxifen at 6 weeks of age. The mean body weight of *Spint2*^LoxP/+^*Prss8*^LoxP/LoxP^CreERT2 mice did not decrease after tamoxifen treatment (Fig. 8a), suggesting that Prss8 is not essential for maintenance of the intestinal epithelium as reported previously^24^. Deletion of *Prss8* gene did not rescue the lethal phenotype caused by postnatal deletion of *Spint2*. After tamoxifen treatment, the body weight loss of *Spint2*^LoxP/LoxP^*Prss8*^LoxP/LoxP^CreERT2 mice was comparable to that of *Spint2*^LoxP/LoxP^*Prss8*^LoxP/+^CreERT2 littermate mice (Fig. 8a) and the intestinal tissues showed similar macroscopic changes (Fig. 8b). We next sought to determine whether Prss8 is required for Epcam cleavage in the presence of Hai-2 ablation in organoids prepared from the small intestines of *Spint2*^LoxP/LoxP^*Prss8*^LoxP/LoxP^CreERT2 mice. In contrast to the observation in vivo, co-deletion of *Prss8* suppressed Epcam cleavage, stabilized claudin-7 levels (Fig. 8c, d) and rescued destruction of crypt organoids caused by Hai-2 ablation (Fig. 8e).

### Matriptase cleaved Epcam in *Spint2*^−/−^ organoids

**Fig. 9:**
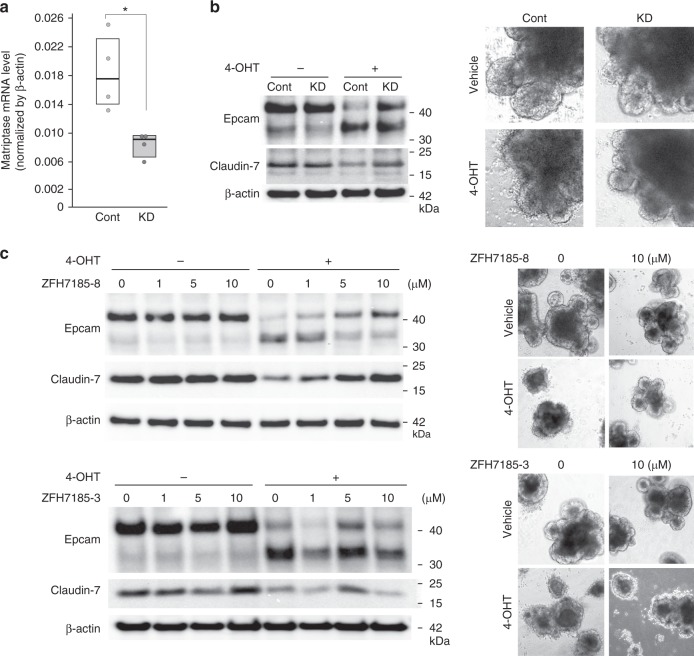
Matriptase is responsible for Epcam cleavage. **a** Knockdown efficacy of matriptase (encoded by *St14* gene) shRNA in organoids from *Spint2*^LoxP/LoxP^CreERT2 mice. RNA was extracted from mock-transfected control (cont) and matriptase shRNA lentivirus vector-transfected (KD) organoids and matriptase mRNA levels were verified by quantitative RT-PCR. The 25th and 75th percentile (boxes) and the median (bold line within the boxes) are plotted. Circle represents the value of each measurement. *p = 0.029 (Student *t*-test; *n*, 4). **b** Control and matriptase-knockdown organoids were treated with 4-OHT (1 μM) or vehicle only (ethanol) for 28 h. Immunoblot analysis of Epcam and claudin-7 (left panel) and representative morphology of organoids (right panel) are shown. **c** Dose-dependent effects of matriptase-selective inhibitor (ZFH7185-8) on Epcam cleavage and claudin-7 in *Spint2*^LoxP/LoxP^CreERT2 organoids with or without 4-OHT treatment (28 h). Hepsin-selective inhibitor (ZFH7185-3) was also used as a control (lower panel). Representative photos of organoids are also shown

It has been reported that Prss8 cannot directly cleave Epcam whereas matriptase can^17^. Thus, we asked whether matriptase was responsible for the enhanced cleavage of Epcam in *Spint2*-deleted intestinal organoids due to dysregulated Prss8-matriptase axis. Transfection of matriptase short hairpin RNA (shRNA) lentiviral vector reduced the matriptase mRNA level by 56%, which partly alleviated the Epcam cleavage in *Spint2*-deleted organoids (Fig. 9a, b). In accordance with this observation, matriptase-selective synthetic inhibitor ZFH7185-8 suppressed the Hai-2 loss-induced Epcam cleavage in a dose-dependent manner, accompanying stabilization of claudin-7 (Fig. 9c). In contrast, hepsin-selective inhibitor ZFH7185-3 did not show notable effects (Fig. 9c).

## Discussion

In this study, we demonstrated that acute ablation of the Hai-2 protein had a lethal effect on mice due to epithelial changes and intestinal dysfunction. Although increased exfoliation of epithelial cells and in turn active cellular proliferation occurred in the intestinal crypts, epithelial regeneration was abnormal such that the intestinal mucosa eventually showed severely disorganized architecture, atrophic villi, continuous exfoliation and tufted formations composed of enterocytes, as well as markedly elongated crypts. Mice carrying the *Spint2* deletion all died within 6 days of initiating tamoxifen treatment.

Congenital *SPINT2* mutations induce a syndromic form of CSD, and the intestinal epithelium of patients suffering from this rare autosomal-recessive disorder shows histological changes similar to those seen in CTE^15,16^. Conventional CTE is caused by a mutation in the *EPCAM* gene wherein abnormal EpCAM function destabilizes EpCAM-associated claudin-7, which eventually results in abnormally enhanced epithelial permeability^15,21,22,25^. Our organoid culture study revealed that Hai-2 loss accelerates Epcam protein cleavage, which was suppressed by the addition of protease inhibitors targeting trypsin-like serine proteases. Therefore, excess cleavage of Epcam by serine proteases could occur in the absence of Hai-2. Evidence indicates that HAI-2/Hai-2 regulates PRSS8/Prss8 in epithelial cells such as enterocytes, which influences matriptase localization and activation in the cells^4,5,10,23^. Given that EpCAM is a matriptase substrate in human enterocytes^17^, abnormal activity of the Prss8-matriptase axis could underpin the enhanced Epcam cleavage that occurred in the absence of Hai-2. Indeed, co-deletion of *Prss8* and *Spint2* in organoids relieved the Epcam cleavage in the absence of Hai-2, and consequently maintained the claudin-7 levels and rescued organoids from subsequent rupture. Moreover, silencing of matriptase in organoids or treatment of the organoids with matriptase-selective synthetic inhibitor alleviated the Hai-2 loss-induced Epcam cleavage. Therefore, Hai-2 tightly regulates Prss8-matriptase axis in the intestinal epithelial cells and dysregulation of this axis results in excess Epcam cleavage by matriptase and destabilization of the Epcam/claudin-7 complex.

Whereas co-deletion of *Prss8* and *Spint2* suppressed Epcam cleavage caused by *Spint2* deletion in organoids, *Spint2*/*Prss8* co-deletion could not rescue the lethal phenotype associated with *Spint2* deletion in mice. This discrepancy may be consistent with recent observations in another CTE model: *Spint2*^−/−^*Prss8*^R44Q/R44Q^ double mutant mice^19^. In the double mutant model, homozygous R44Q mutation of *Prss8* gene resulted in the impaired activation of prostasin zymogen (i.e., zymogen-locked Prss8) and rescued embryonic lethality caused by the *Spint2* deletion, and the intestinal development was normal in *Spint2*^−/−^*Prss8*^R44Q/R44Q^ fetus at E18.5^19^. However, after birth, *Spint2*^−/−^*Prss8*^R44Q/R44Q^ mice showed severe growth retardation and died within a week with severe intestinal abnormalities reminiscence to CTE^19^. Thus, homozygous R44Q mutation in *Prss8* rescued *Spint2* deletion-induced CTE only during the fetal period. Environmental factors, such as intestinal microbial colonization and food intake, may underpin the conflicting observations between mice in vivo after birth and organoids ex vivo that might be equivalent to the fetal intestine. Nonetheless, these lines of evidence raise the question of whether Epcam cleavage is sufficient to induce the severe intestinal phenotype in vivo. In fact, the histological abnormalities observed in the colon mucosa of the *Spint2*-deletion mice in this study were more severe than those reported for *Epcam* mutant mice^21,22^. Moreover, our *Spint2* knockout mice showed epithelial abnormalities in the gallbladder and extrahepatic bile duct, which were not reported in the *Epcam* knockout studies^21,25^. Therefore, the role of Hai-2 in epithelial cells may not be limited to stabilization of the Epcam/claudin-7 complex in the intestine, and Hai-2 may have additional, vital functions for epithelial cells in vivo. HAI-2/Hai-2 immunoreactivity has been observed primarily in the cytoplasm^1,8,10^, and it likely localizes to the endoplasmic reticulum (ER)^9^. HAI-2/Hai-2 may therefore have an important role in regulation of proteolysis in ER and intracellular secretory pathways, which might be required to maintain epithelial integrity in vivo. Further studies to identify proteins interacting with HAI-2/Hai-2 will be required.

Whether Hai-2 loss affects the activity of epithelial sodium channel (ENaC) also remains unclear. Prss8 and matriptase, which are both Hai-2 sensitive, are likely involved in ENaC activation^3,24^, and patients carrying *SPINT2* gene mutations often experience sodium diarrhea. Although the severe effect of spontaneous Hai-2 ablation precluded analysis of ENaC function in our mouse model in vivo, ex vivo intestinal organoids from these mutant mice could provide a tool for future studies to explore the roles of Hai-2 and Prss8 in intestinal ENaC activation. Finally, the effect of Hai-2 loss on intestinal stem cells also awaits analysis. Given that the number of Lgr5-positive intestinal stem cells remained unchanged in *Epcam*-deleted mice^21^, intestinal stem cells may not be a primary target in the *Spint2* deletion-induced phenotype. However, Paneth cells maintain the stem cell niche in intestinal crypts^26^, and the number of Paneth cells was decreased by *Spint2* deletion. Therefore, the maintenance of stem cells may indeed be disturbed in the *Spint2*-deleted intestine.

The conditional *Spint2* knockout mouse described in this report is the second mouse model of Hai-2 deficiency-induced CTE next to *Spint2*^−/−^*Prss8*^R44Q/R44Q^ double mutant mouse recently reported by Szabo and Bugge^19^. Although both models show similar CTE morphology, there are a couple of differences in regard to histological findings. First, mucosal damage, inflammation and apoptotic epithelial cells were more evident in our model compared to the double mutant model. Second, Ki67-positive cells in the intestinal crypts increased significantly in our model, but not in the double mutant model. In addition, abnormalities observed in the extrahepatic biliary epithelium of our mutant mice were not reported in the double mutant mice^19^. These differences may be due to the method of *Spint2* ablation: acute *Spint2* ablation in postnatal period in our model versus genetically *Spint2* null condition in the double mutant model. The strength of the *Spint2*-*Prss8* double mutant model may be an early-onset intestinal failure after birth, which likely mimics the patients of congenital CTE^19^. However, *Prss8* mutation is not reported in the CTE patients. The current conditional *Spint2* knockout mouse model has wild-type *Prss8* and has an advantage in this regard. Another important advantage of the current model is the availability of organoid culture. The intestinal organoids from *Spint2*^LoxP/LoxP^CreERT2 mice reproduces molecular changes induced by Hai-2 deficiency in vitro, providing an indispensable research tool for the study of CTE.

In conclusion, Hai-2 is a vital epithelial protease inhibitor that is needed to maintain the integrity of the intestine and extrahepatic biliary epithelia as well as to stabilize the Epcam/claudin-7 complex. Our *Spint2*-mutant mouse model together with the intestinal organoid culture from these mutant mice would provide a useful tool for detailed studies of HAI-2/Hai-2 function in epithelial cells and the screening of therapeutic compounds for patients carrying *SPINT2* mutations. Successful inhibition of Hai-2 loss-induced Epcam cleavage by an exogenous serine protease inhibitor in an ex vivo organoid culture model could be important for developing treatments for intestinal failure induced by *Spint2* mutations.

## Methods

### Antibodies

The following anti-mouse antibodies were used: anti-Hai-2 goat polyclonal antibody (pAb) (R & D Systems, Minneapolis, MN); anti-Epcam (ProteinTech., Rosemont, IL), anti-claudin-7 (Thermo Fisher Scientific, Waltham, MA), anti-mouse β-catenin (Sigma, St. Louis, MO), anti-cleaved caspase-3 (Cell Signaling, Boston, MA), and anti-lysozyme (AVIVA Systems Biology, San Diego, CA) rabbit pAbs; anti-Ki67 rabbit monoclonal antibody (mAb) (Abcam, Cambridge, UK); anti-E-cadherin goat pAb (R & D Systems); anti-claudin-2 and anti-ZO-1 rabbit pAbs (Thermo Fisher Scientific); anti-β-actin mouse mAb (Sigma) and anti-ssDNA rabbit pAb (Dako, Carpinteria, CA).

### Generation of *Spint2* conditional knockout mice

All of the animal work was performed under the approval of the University of Miyazaki Animal Research Committee, in accordance with international guidelines for biomedical research involving animals. The required portions of the mouse *Spint2* gene were amplified by PCR from a mouse (C57BL/6) genomic bacterial artificial chromosome library and subcloned into a pBluescript II SK + phagemid vector (Agilent Technologies, Palo Alto, CA), which was used to construct a targeting vector. LoxP sites were inserted into exons 2 and 6 to delete all transcript variants of the *Spint2* gene^27,28^ following activation of Cre recombinase. A neomycin resistance gene (*neo*) cassette for positive selection was inserted into intron 6 and was flanked by short flippase recognition target (FRT) sites. The targeting vector also contained a diphtheria toxin expression cassette for negative selection and had an *Mfe*I site for screening by Southern blotting. The targeting vector was linearized with *Sac*II and transfected into C57BL/6 strain ES cells by electroporation. Correctly targeted ES clones were selected using G418 (Sigma). Two independently targeted ES clones (ES16 and ES89) were obtained and microinjected into morulae of ICR mice. The resulting chimeras were mated with C57BL/6 mice. Germline transmission of the targeted allele was detected by progeny coat color and PCR. FLPeR mice^29^ were purchased from The Jackson Laboratory (Bar Harbor, ME) and the mutant mice were cross-mated for more than nine generations with C57BL/6 mice (Charles River Laboratories, Portage, MI). Then, *Spint2*^LoxP/+^/*neo* + mice were crossed to the B6-background FLPeR mice to remove the *neo* cassette. Heterozygous offspring (*Spint2*^LoxP/+^) were crossed to produce homozygous mutant offspring (*Spint2*^LoxP/LoxP^). *Spint2*^LoxP/LoxP^ mice were further crossed with ROSA26-CreERT2 mice (The Jackson Laboratory) to generate ROSA26-CreERT2 mice with the floxed *Spint2* gene (hereafter *Spint2*^LoxP/LoxP^CreERT2). To activate the CreERT2 recombinase, 6-week-old male mice were treated by intraperitoneal injection of 50 mg (135 μmol)/kg tamoxifen (Sigma) dissolved in corn oil for 3 consecutive days. Successful DNA rearrangement and loss of *Spint2* mRNA were validated by genomic PCR and revere-transcription (RT)-PCR using DNA and total RNA, respectively, extracted from the tissues. The primers used for the validation described above are shown in Supplementary Fig. 1 and Supplementary Table 2. The site of each primer for genomic PCR is indicated in Fig. 1a. Unless otherwise indicated, data from the ES16 mouse line are presented.

To generate *Spint2* and *Prss8* double conditional knockout mice, *Spint2*^LoxP/+^CreERT2 mice were crossed with *Prss8*^LoxP/+^ mice^30^. Primer sequences for genotyping of the *Prss8* mutant mice are shown in Supplementary Table 2.

### Histological analysis

For histological analysis, mice tissues were fixed in 4% formaldehyde in phosphate-buffered saline (PBS) and embedded in paraffin. Four-micrometer-thick sections were stained with hematoxylin and eosin (HE). Villus length and crypt depth were measured using Olympus cellSens imaging software (v1.15). To count the number of Paneth cells, ten randomly selected areas were photographed at 200× magnification from each mouse small intestine. Then, two independent investigators counted the number of Paneth cells per crypt. To count the number of goblet cells, large intestinal tissues were stained with alcian-blue, and ten randomly selected areas were photographed at 200× magnification from each mouse. Then, two independent investigators counted the number of goblet cells per crypt.

### Immunohistochemical staining of mouse tissue sections

Mice were sacrificed at the indicated time point, autopsied and tissues were fixed with 4% formaldehyde in PBS. Immunohistochemistry of formalin-fixed, paraffin-embedded tissue sections was performed as described previously^31,32^ except for Hai-2 and ssDNA. Briefly, tissue sections were processed for antigen retrieval by autoclaving for 5 min at 121 °C in 1 mM EDTA pH 8.0 for cleaved caspase-3 or in 10 mM citrate buffer pH 6.0 for other antibodies, followed by treatment with 3% H2O2 in PBS for 10 min. After blocking in 3% bovine serum albumin in PBS, the sections were incubated with primary antibodies for 16 h at 4 °C: anti-Ki-67 (1:100 dilution), anti-cleaved caspase-3 (1:300 dilution), anti-Lysozyme (1:50 dilution), anti-Epcam (1:250 dilution), anti-claudin-7 (1:100 dilution), and anti-β-catenin (1:2000 dilution). After washing with PBS, the sections were incubated with Envision^TM^ labeled polymer reagents (DAKO) for 30 min at room temperature (RT). For anti-Hai-2 pAb (1:250 dilution), Ventana Discovery system (Ventana Medical System, Tucson, AZ) was used according to the manufacturer’s instructions. For immunostaining of ssDNA, deparaffinized tissue sections were incubated with PBS containing 0.2 mg/mL saponin (Nacalai Tesque, Kyoto, Japan) and 20 μg/mL Proteinase K (Nacalai Tesque) at RT for 20 min. After washing the slides in distilled water, sections were heated in preheated 50% formamide in distilled water at 56 °C for 20 min, followed by treatment with 3% hydrogen peroxide for 10 min and washing in PBS. After blocking in 3% non-fat dry milk for 20 min at 37 °C, the sections were incubated with anti-ssDNA (1:700 dilution) in 1% non-fat dry milk at RT for 30 min. The sections were then washed in PBS and incubated with Envision-labeled polymer reagent (DAKO) for 30 min at RT. The reaction was revealed with nickel, cobalt-3,3’-diaminobenzidine (Thermo Fisher Scientific) and counterstained with hematoxylin.

### Intestinal permeability assay

To assess intestinal barrier function, an intestinal permeability assay was performed as previously described^31^. Briefly, 250 μg/g body weight fluorescein isothiocyanate (FITC)-dextran (4 kDa; Sigma) was administered to the mice by gavage 45 h after starting tamoxifen treatment. Three hours after administration of FITC-dextran, blood samples were obtained from the right ventricle, and EDTA-containing plasma samples were collected by centrifugation at 960 × *g* for 20 min at 4 °C. Plasma (75 μL) was diluted in 250 μL PBS, and fluorescence was quantified using a fluorometer (DTX800 Multimode Detector; Beckman Coulter, Fullerton, CA) with excitation and emission wavelengths of 485 and 535 nm, respectively. FITC-dextran concentrations were determined from standard curves generated by serial dilution of FITC-dextran.

### Camostat mesilate administration

Camostat mesilate (Ono Pharmaceutical, Osaka, Japan) was administered to the mice through addition to the diet or by gavage, or intraperitoneal injection. For dietary administration, mice were given a diet containing 0.1% camostat mesilate three days before starting tamoxifen treatment until termination. For gavage or intraperitoneal injection, mice were treated with camostat mesilate solution (30 μg/μL water; 200 mg/kg) every 12 h beginning at the same time as tamoxifen treatment was initiated.

### Matriptase-selective synthetic inhibitor

General procedure for the preparation of α-ketobenzothiazole (kbt) serine protease inhibitors for HGFA, matriptase or another TTSP namely hepsin has been reported previously^33^ and described in Supplementary Methods. Florescent kinetic enzyme inhibitor assays were performed to analyze the selectivity of the kbt inhibitors using either recombinant catalytic domains of HGFA^34^, matriptase (#3946-SEB, R&D Systems) or hepsin (#4776-SE, R&D Systems) in black 384 plates (Corning #3575, Corning, NY) (Supplementary Methods). Two kbt inhibitors, ZFH7185-8 and -3, were prepared (Supplemental Methods). ZFH7185-8 is Fmoc-Gly-Arg-kbt that is selective for matriptase (matriptase Ki, 0.13 nM; hepsin Ki, 831 nM), and ZFH7185-3 is Fmoc-Asn-Arg-kbt that is selective for hepsin (matriptase Ki, 106 nM; hepsin Ki, 1.7 nM) (Supplementary Methods).

### Intestinal organoid culture

Isolation of intestinal crypts and organoid culture were performed as described previously^35^. Briefly, small intestine tissue isolated from 3- to 4-week-old male mice was washed with cold PBS and minced into 3 mm pieces. After washing again with cold PBS, the fragments were incubated for 30 min at 4 °C in PBS containing 2 mM EDTA, followed by centrifugation (400 *×* *g*, 3 min). Then, the fragments were suspended in cold PBS and passed through a 70-μm cell strainer (BD Biosciences, Bedford, MA). Isolated crypts were collected by centrifugation and further dissociated into single cells by Accumax (Innovative Cell Technologies, San Diego, CA) treatment for 5 min at 37 °C. For 3-D organoid culture, the dissociated single cells were resuspended in medium (Advanced DMEM/F12; Invitrogen, Tokyo, Japan) supplemented with 50 ng/mL EGF (Peprotech, Rocky Hill, NJ), 500 ng/mL R-Spondin1 (R&D systems), 100 ng/mL Noggin (Peprotech), 10 μM Y27632 (Wako, Osaka, Japan) and 1 μM Jagged-1 (AnaSpec, San Jose, CA), and seeded on Matrigel (60 μL/well; BD Biosciences) in 12-well plates, which were incubated overnight at 37 °C. After removing floating dead cells and medium, viable cells attached to the Matrigel were covered with 80 μL Matrigel and overlaid with complete culture media to resume 3-D culture. After the organoids formed, they were cultured with or without 1 μM 4-OHT (Sigma) in the presence or absence of aprotinin (Sigma) or camostat mesilate. To recover organoids from the Matrigel matrix and prepare sections, BD Cell Recovery Solution (BD Biosciences) was used according to the manufacturer’s instructions. Then the organoids were centrifuged (300 × *g*, 5 min) at 4 °C, formed into gels using iPGell (GenoStaff, Tokyo Japan) and fixed in 4% paraformaldehyde in PBS followed by embedding in paraffin. Sections (3 μm) were prepared and used for hematoxylin & eosin (HE) staining and immunostaining.

### Analysis of junctional proteins in organoids

Organoids were extracted with RIPA buffer (Nacalai Tesque) and used for immunoblotting analysis. The membranes were incubated with antibodies against Epcam (1:1000 dilution), claudin-7 (1:1000 dilution), claudin-2 (1:1000 dilution), E-cadherin (1:1000 dilution), and β-actin (1:1000 dilution) at 4 °C overnight. To detect ZO-1, immunofluorescence staining was performed as described previously^31^. Briefly, organoids were fixed with 4% paraformaldehyde in PBS for 15 min, followed by blocking for 30 min with 5% normal goat serum (Dako) at RT. Then the organoids were incubated with anti-ZO-1 pAb (1:25 dilution) for 16 h at 4 °C. After washing, the cells were incubated for 30 min at RT with Alexa Fluor 488-conjugated goat anti-rabbit IgG (Invitrogen). Then, the organoids were counterstained with 4,’6-diamino-2-phenylindole (Sigma), and examined with an Axio Imager A2 (Carl Zeiss MicroImaging, Tokyo, Japan). To validate Epcam and claudin-7 mRNA expression, RT-PCR was performed using total RNA extracted from organoids. Primer sequences are shown in Supplementary Table 2. Full size immunoblots and gels for all experiments are shown in Supplementary Fig. 6.

### Silencing of matriptase in organoids

For the silencing of matriptase (encoded by *St14* gene) expression, shRNA against *St14* was inserted into pLenti4/BLOCK-iT (Life Technologies Japan). Generation of the lentiviral particles was performed according to the manufacturer’s instructions. Lentiviral particles were infected as previously described^35^. The target sequence was 5′-GCGCTTCAAACTCTTCTATCT-3′. To confirm the efficiency of *St14* gene knockdown, real-time RT-PCR was performed in a Thermal Cycler Dice Real Time System II (Takara Bio) using the SYBR Premix Ex Taq II (Takara Bio). The primer sequences are shown in Supplementary Table 2.

### Statistical analysis

Statistical analysis was done using StatView 5.0 (SAS, Cary, NC, USA). Comparisons between two unpaired groups were done with the Mann–Whitney *U*-test, Student’s *t*-test or two-way repeated-measures analysis of variance (ANOVA). Significance was set at *p* < 0.05.

## Supplementary information

Supplementary Information

Description of Additional Supplementary Files

Supplementary Movie 1

Supplementary Movie 2

Supplementary Movie 3

## Data Availability

All data generated or analyzed during this study are included in this published article and its Supplementary Information files.
