# Supplementary material for: Hepatocyte growth factor activator inhibitor-2 stabilizes Epcam and maintains epithelial organization in the mouse intestine
Source: Commun Biol. 2019 Jan 4;2:11. doi: 10.1038/s42003-018-0255-8 (PMC6320337; doi:10.1038/s42003-018-0255-8)
Supplement: Supplementary file 1 — Supplementary Information [file 42003_2018_255_MOESM1_ESM.pdf]

Supplementary Figures

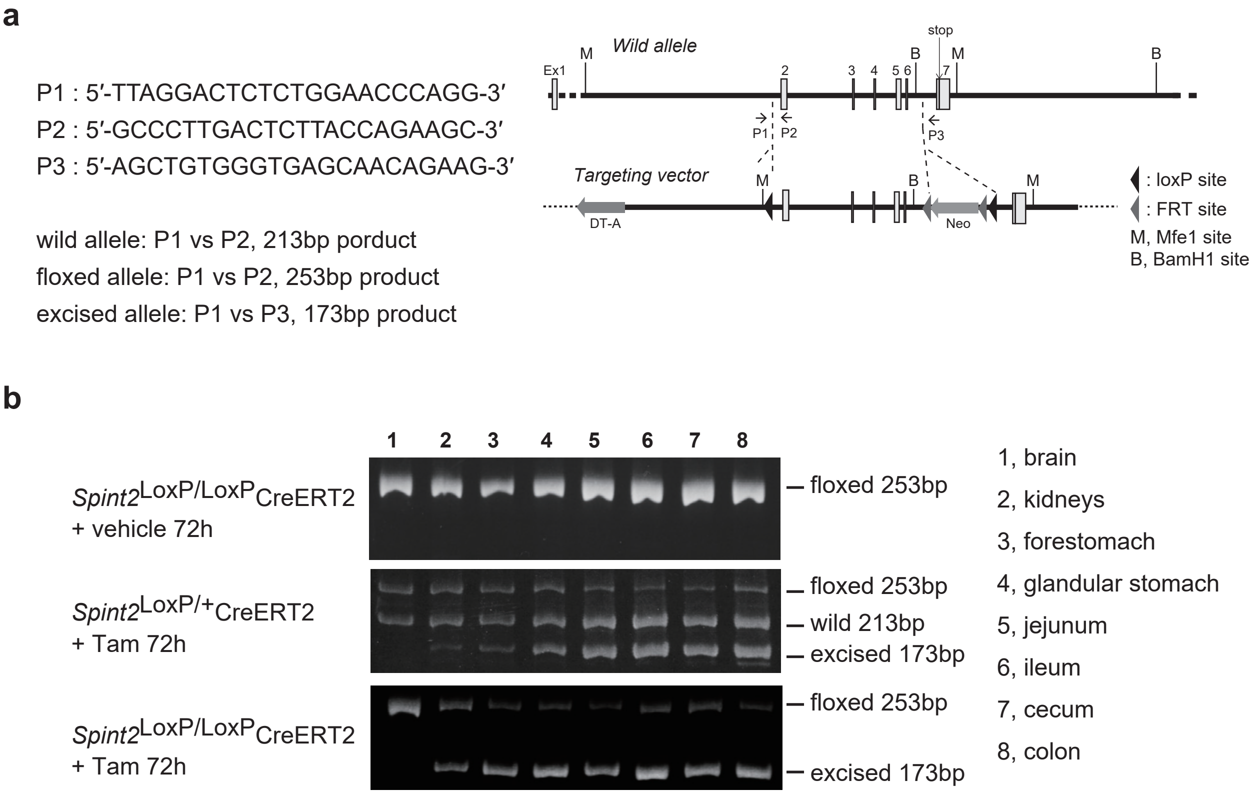

Supplementary Figure 1. Genomic PCR of *Spint2*. **a** Sequences and positions of primers for the validation of *Spint2*. **b** Results of genomic PCR of various organs 72 h after starting tamoxifen (Tam) treatment.

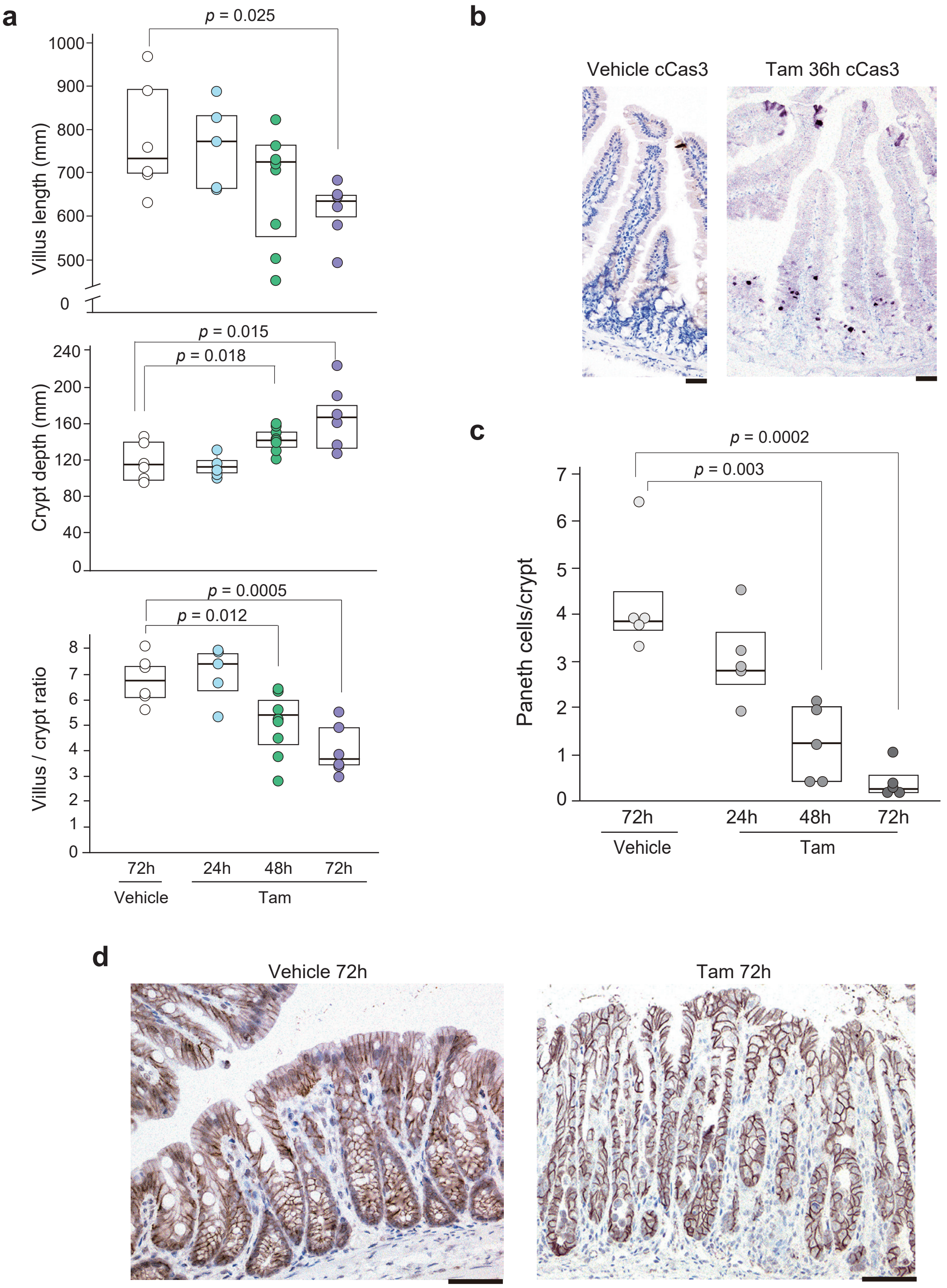

Supplementary Figure 2: Effects of *Spint2* deletion on the mucosal organization of murine intestine. **a** Decreased villus/crypt ratio in *Spint2*-deleted proximal small intestine. Data from mice treated with vehicle only (n, 6) or with tamoxifen (Tam) for 24h (n, 5), 48h (n, 8) or 72h (n, 6) are shown (*p* value; Mann-Whitney U test). **b** Immunohistochemistry of cleaved caspase 3 (cCas3). A section of proximal small intestine 36 h after tamoxifen (Tam) treatment was immunostained and compared with vehicle-treated control mouse mucosa. Bars, 50 µm. **c** Decreased number of Paneth cells in the crypt base of the small intestine. N=5 for each group and 10 crypts were analyzed for each mouse (*p* value; Mann-Whitney U test). **d** Immunohistochemistry for β-catenin in the large intestine. Nuclear translocation of β-catenin was not enhanced by *Spint2* deletion. Bar, 50 µm. In **a** and **c**, The 25th and 75th percentile (boxes) and the median (bold line within the boxes) are plotted. Circles represent the value of each case.

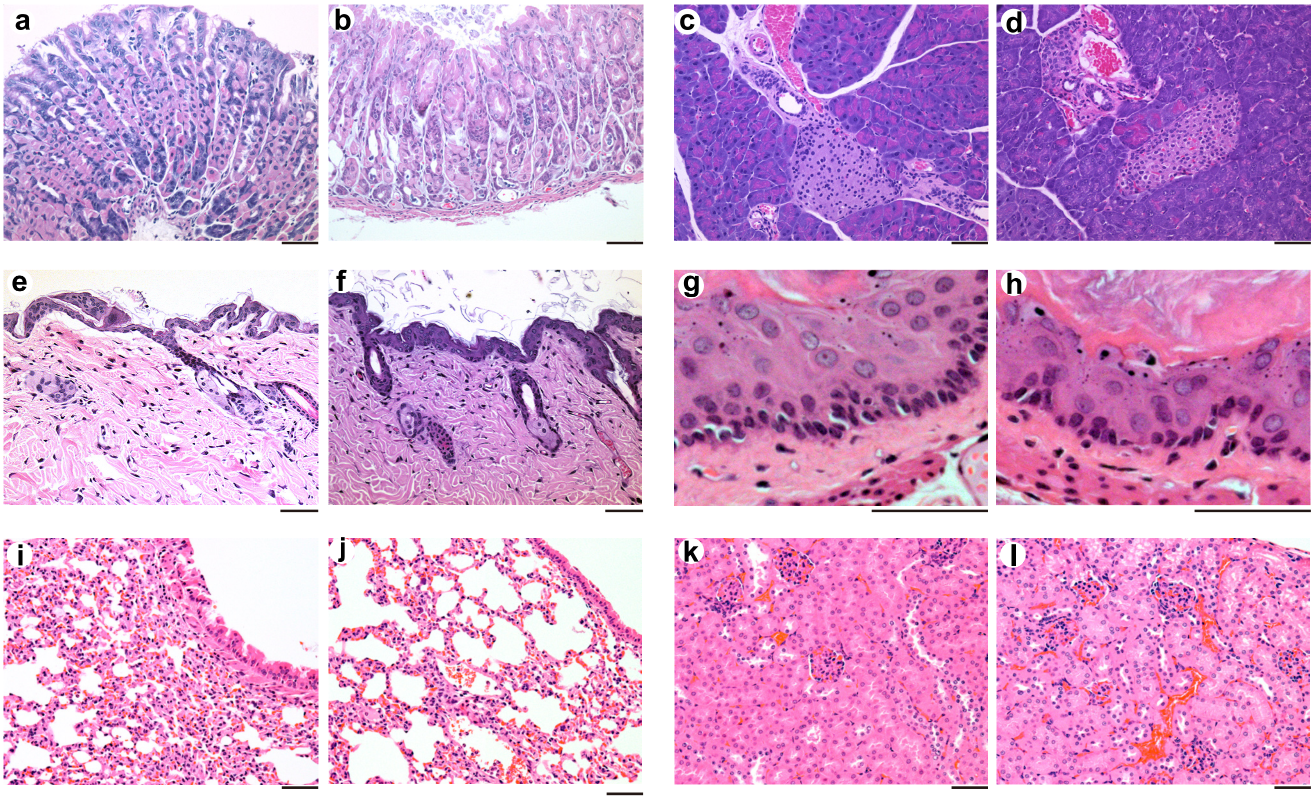

Supplementary Figure 3. Histology of extra-intestinal tissues in mutant mice. Representative photos of HE sections of epithelial tissues from corn oil (vehicle)-treated (**a**, **c**, **e**, **g**, **i**, **k**) or tamoxifen-treated (**b**, **d**, **f**, **h**, **j**, **l**) *Spint2*$^{\mathrm{LoxP/LoxP}}$/CreERT2 mice are shown. **a** and **b**, glandular stomach; **c** and **d**, pancreas; **e** and **f**; skin; **g** and **h**; esophagus; **i** and **j**, bronchial epithelium and alveolar tissue of the lung; **k** and **l**, kidney. Bars, 50 μm.

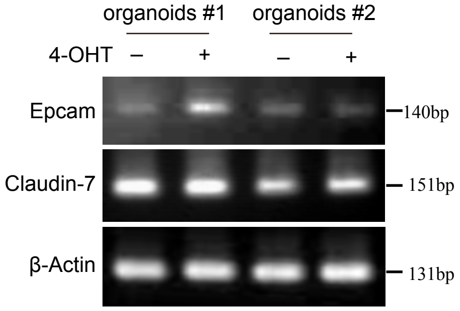

Supplementary Figure 4. RT-PCR analysis of Epcam and claudin-7 expression in organoids. Intestinal organoids from *Spint2*$^{\text{LoxP/LoxP}}$/CreERT2 mouse were treated with or without 4-OHT for 24 h. Results of two independent experiments are shown.

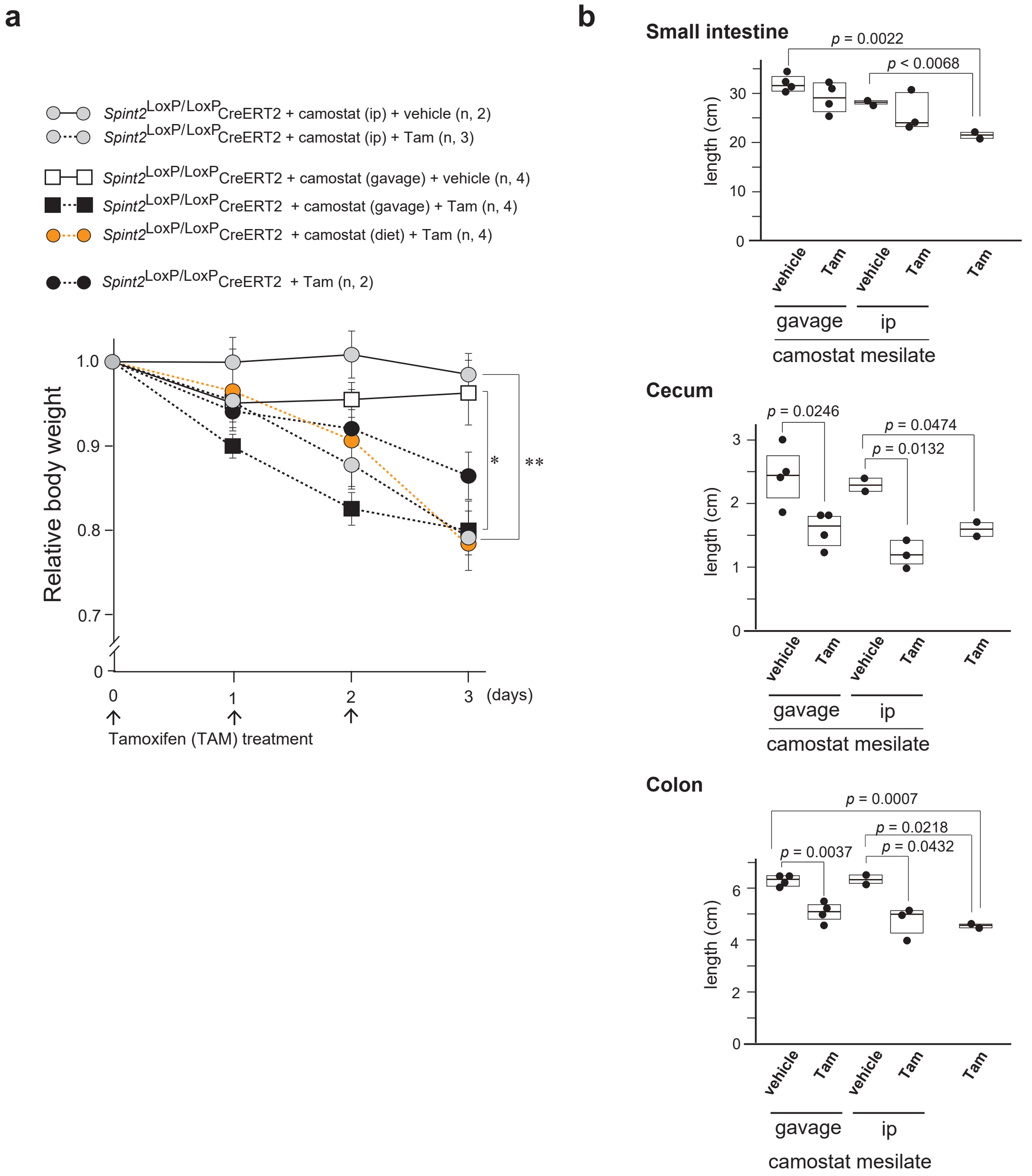

Supplementary Figure 5. Effects of camostat mesilate administration on tamoxifen-treated *Spint2*$^{LoxP/LoxP}$ CreERT2 mice. **a** The effect of camostat mesilate on body weight gain in mice with spontaneous *Spint2* deletion. Six-week-old male mice were treated with tamoxifen (Tam) or vehicle for 3 days with or without administration of camostat mesilate by intraperitoneal injection (ip) (200 mg kg^{-1}, every 12 h beginning at the same time as Tam treatment), by gavage (200 mg/kg, every 12 h beginning at the same time as Tam treatment), or dietary (0.1% in a diet, beginning at 3 days before starting Tam treatment). *, $p = 0.00039$; **, $p < 0.0001$, two-way repeated-measures ANOVA. Error bar, SE. **b** Effect of camostat mesilate on the length of small intestine, cecum and colon 72 h after Tam or vehicle treatment. The 25th and 75th percentile (boxes) and the median (bold line within the boxes) are plotted. Circles represent the value of each case.

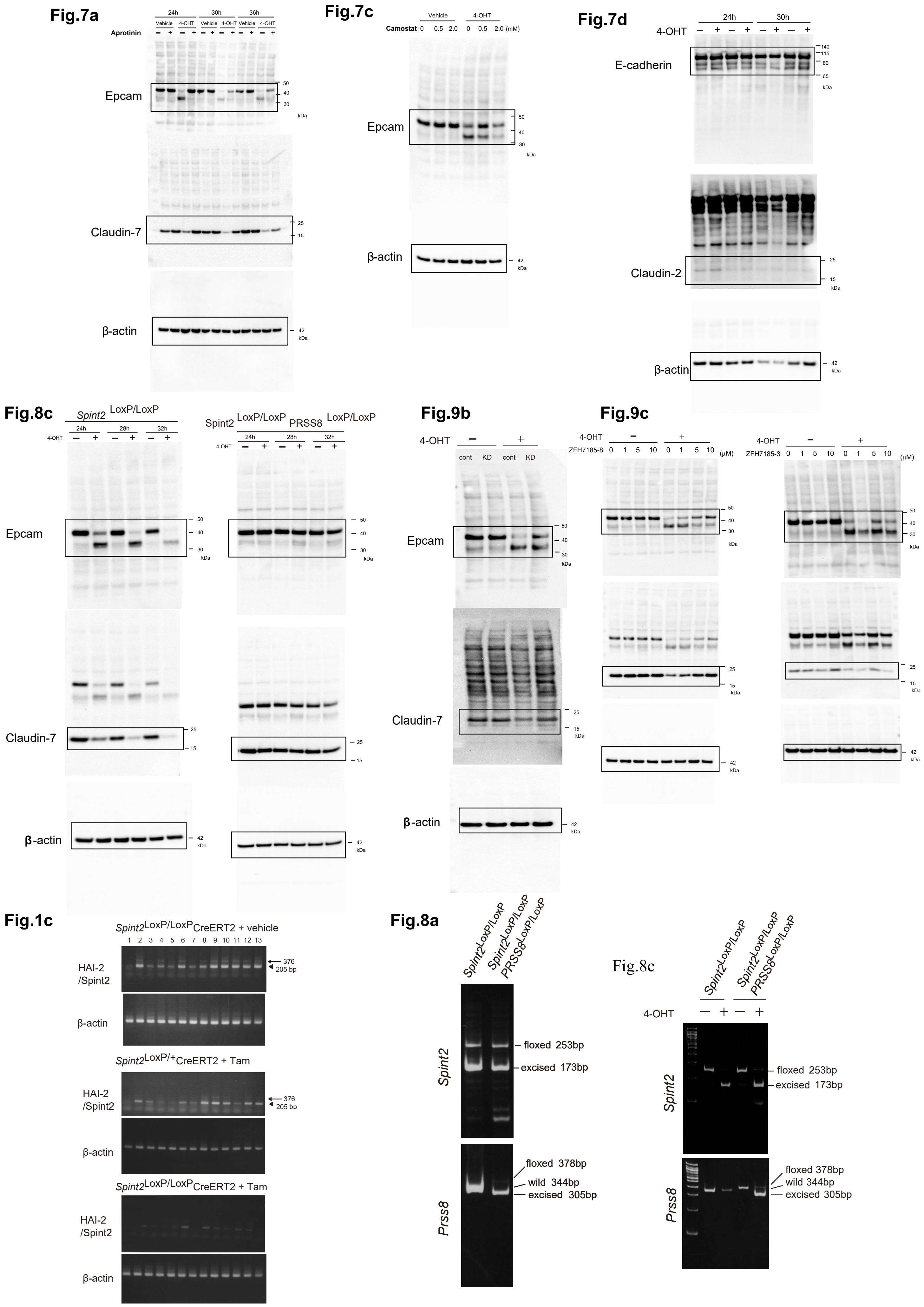

Supplementary Figure 6. Original immunoblots and full length gels of all experiments in the indicated figures.

Supplementary Tables

Supplementary Table 1. Effects of *Spint2* ablation on survival and intestinal length (3 days after treatment) in mutant mice (6 weeks old) derived from ES89 clone

Mice	Treatment (n)	*Spint2* genotype	Survival*	Length of intestine, mean ± SE (n)		
				Small intestine	Cecum	Large intestine
Spint2$^{LoxP/LoxP}$CreERT2	Vehicle (2)	+/+	100% (6/6)	35.5 ± 0.79 (6) [a]	2.28 ± 0.10 (6) [c]	6.56 ± 0.15 (6) [e]
Spint2$^{LoxP/LoxP}$	Tam (4)					
Spint2$^{LoxP/+}$CreERT2	Tam (5)	+/−	100% (5/5)	33.6 ± 1.12 (5) [b]	2.14 ± 0.18 (5) [d]	6.44 ± 0.16 (5) [f]
Spint2$^{LoxP/LoxP}$CreERT2	Tam (4)	−/−	0% (0/4)	29.5 ± 0.57 (4)	1.23 ± 0.14 (4)	5.10 ± 0.20 (4)

*, survival ratio 5 days after starting tamoxifen (Tam) or vehicle treatment. a; p =0.0004, b; p=0.0134, c; p =0.0003, d; p =0.0094, e; p =0.0005, f; p =0.0015 compared to *Spint2*$^{LoxP/LoxP}$CreERT2 + Tam group (Student T-test).

Supplementary Table 2. Primer sequences for RT-PCR and *Prss8* genotyping

Target (RT-PCR)	Forward	Reverse	Size
HAI-2 (*Spint2*)	5′-TCGCCTTGGTAGCTTCGCTG (P4 in Figure 1A)	5′-GTGACGGCCTTCGGGACACA (P5 in Figure 1A)	376bp (minor isoform) 205bp (major isoform)
Epcam (*Epcam*)	5′-CTTCAAGAGGCGTTCACATC	5′-ACATCAGCTATGTCCACGTC	140bp
Claudin-7 (*Cldn7*)	5′-GGTGTTGGGCTTCTTAGCCATG	5′-AGGAACATGCTACCAAGGCAGC	151bp
Matriptase (*St14*)	5′-CACGAATGATGTGTGTGGGTTTC	5′-CCTGGAACATTCGCCCATCT	105bp
β-Actin (*Actb*)	5′-TGACAGGATGCAGAAGGAGA	5′-GCTGGAAGGTGGACAGTGAG	131bp

Prss8 genotyping primers	Sequence	Target
Prss8 P1	5′-CTGTAGCTGCCTGTACAACATTA	Wild allele (P1 vs P2): 344bp<br>Floxed allele (P1 vs P2): 378bp<br>Excised allele (P2 vs P3): 305bp
Prss8 P2	5′-CAGGAAGCATAGGTAGAAGTCAGAG	
Prss8 P3	5′-TAAAGGTCCAGTGGTAAGGAGTGAG	

Supplementary Methods

Preparation of α-ketobenzothiazole (kbt) serine protease inhibitor compounds

Arg (Mtr)-kbt HCl (**1**, 0.02 mmol) was coupled with Fmoc-L-amino acid (Fmoc-X) with standard protecting groups (**2**, 0.02 mmol) using 1-[Bis(dimethylamino)methylene]-1H-1,2,3-triazolo[4,5-b]pyridinium 3-oxid hexafluorophosphate (0.02 mmol; Sigma) as coupling reagent in presence of iPr2NEt (0.1 mmol) in N,N-dimethylformamide (0.5 mL) under a nitrogen atmosphere. After stirring for 4h at room temperature, the reaction was concentrated in vacuo and deprotection of the crude product was accomplished by stirring in 0.3 mL of a trifluoroacetic acid (TFA)-thioanisole-water mixture (95:2.5:2.5) for 4-5 h. After concentrating in vacuo, the crude material was dissolved in dimethyl sulfoxide (DMSO) and purified using reverse phase high performance liquid chromatography (0.05% TFA/acetonitrile/water gradient). The pure fractions were pooled and lyophilized to give the dipeptides, Fmoc-XR-kbt (**3**) as white powders.

ZFH7185-3 is Fmoc-Asn-Arg-kbt, (9H-fluoren-9-yl)methyl N-[(1S)-1-{[(2S)-1-(1,3-benzothiazol-2-yl)-5-carbamimidamido-1-oxopentan-2-yl]carbamoyl}-2-carbamoylethyl]carbamate (**3a**). Yield (80%). 1H NMR (400 MHz, METHANOL-*d*4) δ ppm 8.15 - 8.25 (m, 1 H), 8.03 - 8.16 (m, 1 H), 7.79 (d, *J*=7.43 Hz, 2 H), 7.52 - 7.69 (m, 4 H), 7.21 - 7.44 (m, 4 H), 5.64 - 5.78 (m, 1 H), 4.53 - 4.67 (m, 1 H), 4.29 (d, *J*=7.43 Hz, 2 H), 4.12 -4.24 (m, 1 H), 3.17 - 3.30 (m, 2 H), 2.68 - 2.85 (m, 2 H), 2.10 - 2.27 (m, 2 H), 1.63 - 1.96 (m, 4 H). LCMS (ESI+), calculated m/z 627.2, found 628.4 (M+H$^+$). ZFH7185-8 is Fmoc-Gly-Arg-kbt, (9H-fluoren-9-yl)methyl N-({[(2S)-1-(1,3-benzothiazol-2-yl)-5-carbamimidamido-1-oxopentan-2-yl]carbamoyl}methyl)carbamate (**3b**). Yield (75%). ^{1}H NMR (400 MHz, METHANOL-*d*4) δ ppm 8.02 - 8.26 (m, 2 H), 7.50 - 7.84 (m, 6 H), 7.19 - 7.46 (m, 4 H), 5.70-5.81 (m, 1 H), 4.17 - 4.39 (m, 3 H), 3.72 - 3.94 (m, 2 H), 3.24 (br. s., 2 H), 2.18 (br. s., 1 H), 1.65 - 1.87 (m, 3 H). LCMS (ESI+), calculated m/z 570.2, found 571.4 (M+H$^+$).

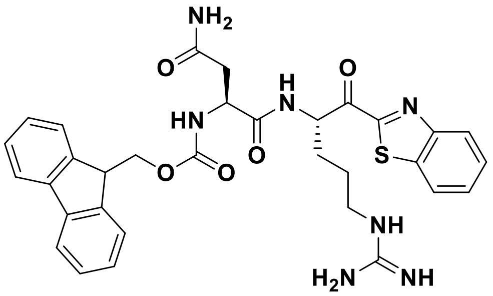

(3a) ZFH7185-3

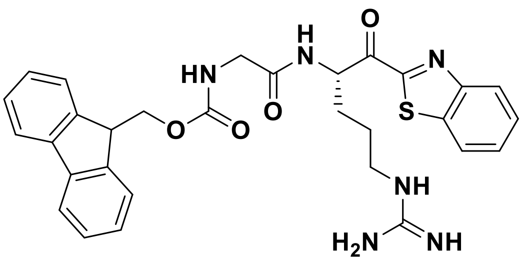

(3b) ZFH7185-8

Fluorescent Kinetic Enzyme Inhibitor Assays of HGFA, matriptase, and hepsin

Inhibitors were serially diluted to 11 concentrations (0-20 µM final concentration in reaction) in DMSO (2% DMSO final concentration) and then mixed with either recombinant catalytic domains of HGFA,[34] matriptase (#3946-SEB, R&D Systems) or hepsin (#4776-SE, R&D Systems) in black 384 well plates (Corning#3575, Corning, NY). Prior to the assay, activation of hepsin was performed as follows: Recombinant hepsin (10 µg, 0.44mg/mL) was diluted to 2.4 µM in TNC buffer (25 mM Tris, 150 mM NaCl, 5 mM $CaCl_2$, 0.01% Triton X-100, pH 8) and incubated at 37°C. After twenty-four hours, the hepsin was diluted in glycerol to 50%. This stock hepsin (1.2 µM) was stored in a -20°C freezer and diluted in TNC buffer for use in assays.

The final assay concentration for HGFA, matriptase and hepsin 7.5 nM, 0.2 nM, and 0.3 nM, respectively in TNC buffer. After thirty minutes incubation at room temperature, Boc-QLR-AMC fluorogenic peptide substrate (#ES011, R&D Systems) (K_m = 37 µM) was added to the HGFA assays and Boc-QAR-AMC (#ES014, R&D Systems) was added to the matriptase (K_m = 93 µM) and hepsin (K_m = 156 µM) assays. The final substrate concentrations for all assays were at the K_m for the respective enzymes. Changes in fluorescence (excitation at 380 nm and emission at 460 nm) were measured at room temperature over time in a Biotek Synergy 2 plate reader (Winnoski, VT). Using GraphPad Prism version 6.04 software program, (GraphPad Software, San Diego, CA, www.graphpad.com), a four-parameter curve fit was used to determine the inhibitor IC_{50}s from a plot of the mean reaction velocity versus the inhibitor concentration. The IC_{50} values represent the average of three separate experimental determinations. K_i* values were calculated using the Cheng and Prusoff equation (K_i= $IC_{50}/(1+[S]/K_m)$).

Compound	Matriptase K_i* (nM)	Hepsin K_i* (nM)	HGFA K_i* (nM)	Matriptase Selectivity	Hepsin Selectivity
**ZFH7185-8** (Fmoc-Gly-Arg-kbt)	0.13	831	9375	6392	-
**ZFH7185-3** (Fmoc-Asn-Arg-kbt)	106	1.7	4771	-	62
