# Supplementary material for: Hepatocyte growth factor activator inhibitor-2 stabilizes Epcam and maintains epithelial organization in the mouse intestine
Source: Commun Biol. 2019 Jan 4;2:11. doi: 10.1038/s42003-018-0255-8 (PMC6320337; doi:10.1038/s42003-018-0255-8)
Supplement: Supplementary file 2 — Description of Additional Supplementary Files [file 42003_2018_255_MOESM2_ESM.docx]

**Description of Additional Supplementary Files**

**File Name**: Supplementary Movie 1

**Description**: Time lapse movie of 4-OHT-treated organoids from small intestine of *Spint2*^LoxP/LoxP^/CreERT2 mouse.

**File Name**: Supplementary Movie 2

**Description**: Time lapse movie of 4-OHT-treated organoids from small intestine of *Spint2*^LoxP/LoxP^CreERT2 mouse different from that of Supplementary Movie 1.

**File Name**: Supplementary Movie 3

**Description**: Time lapse movie of vehicle-treated control organoids from small intestine of *Spint2*^LoxP/LoxP^CreERT2 mouse.
